# Intention to Switch to Smokeless Tobacco Use among South African Smokers: Results from the 2007 South African Social Attitudes Survey

**DOI:** 10.1371/journal.pone.0095553

**Published:** 2014-04-17

**Authors:** Olalekan A. Ayo-Yusuf, Israel T. Agaku

**Affiliations:** 1 School of Health Systems and Public Health, University of Pretoria, Pretoria, South Africa; 2 Office of the Director, School of Oral Health Sciences, University of Limpopo, MEDUNSA campus, Pretoria, South Africa; 3 Center for Global Tobacco Control, Department of Social and Behavioral Sciences, Harvard School of Public Health, Boston, Massachusetts, United States of America; The University of Auckland, New Zealand

## Abstract

**Background:**

Some smokeless tobacco products (SLT) have been shown to be associated with only a fraction of the risks of cigarettes. This study assessed South African smokers’ interest in switching to a hypothetical reduced harm SLT product.

**Methods:**

The 2007 South African Social Attitudes Survey was analysed for 678 exclusive cigarette smokers. Respondents were asked about their perceptions about relative harm of snuff compared to cigarettes, and their interest in switching to snuff if informed it was 99% less harmful than cigarettes.

**Results:**

About 49.7% of exclusive cigarette smokers believed that snuff was equally as harmful as cigarettes; 12.9% thought snuff was more harmful; 5.7% thought snuff was less harmful; while 31.8% did not know if there was a difference in harm between snuff and cigarettes. Approximately 24.2% of exclusive cigarette smokers indicated interest in switching to snuff, with significantly greater interest observed among those exposed to 100% smoke-free work environment. Interest in switching was highest (34.7%) among smokers who believed *a priori* that using snuff was more harmful than cigarettes, and lowest (14.5%) among those who did not know if there was a difference in harm. In a multi-variable adjusted logistic regression model, this latter group remained less likely to be interested in harm reduction switching (adjusted odds ratio = 0.42; 95% CI: 0.19–0.91).

**Conclusion:**

About a quarter of smokers indicated interest in harm reduction switching to snuff. SLT products have a potential role in reducing the harm from smoking in South Africa, but only if they are not used to circumvent smoke-free laws that have been associated with reduced smoking.

## Introduction

Smokeless tobacco (SLT) is a small category within the South African tobacco market, with the majority of users being black females of low socio-economic status. [1] Unlike India and several Western countries where most SLT products are used orally (e.g., chewing tobacco and Swedish-style snus), most commercial and traditional SLT products in South Africa are used nasally. A recent study showed that the levels of carcinogenic tobacco-specific nitrosamines in traditional South African SLT products are up to 19 times higher than those in Swedish-style snus. [2] Although low-nitrosamine, Swedish-style snus was introduced into the South African SLT market in 2005 for situational use - ‘where you can’t smoke’ and as a reduced risk product, [3] uptake of the product has remained low in South Africa.

“First, do no harm” remains one of the guiding principles in medicine and public health. However, the definition of what constitutes population-level “harm” regarding use of modified-risk tobacco products remains controversial in the tobacco control community. Proponents of harm reduction hold that it is paternalistic and unethical for public health authorities to fail to provide comprehensive information to smokers that SLT products are relatively less harmful than cigarettes.[4]–[6] On the other hand, the counter-argument is made that no tobacco product is safe, and that it is harmful to promote a product that has significant potential for addiction, abuse liability, and disease, including oral cancer. [7], [8] Hence the sale and use of SLT products has remained banned in several regions including Australia and parts of Europe. [9], [10] Nonetheless, based on scientific evidence on relatively lower content of tobacco carcinogens in certain SLT products compared to combustible tobacco products, and also evidence from countries such as Sweden where low-nitrosamine snus has been widely used as a harm reduction strategy, the potential role of SLT products in reducing smoking-attributable morbidity and mortality must be acknowledged. [11], [12] However, this potential benefit of SLT can only be optimal in an environment where the use of combustible tobacco products is at a minimum, with SLT being used in lieu of cigarettes and other combustible tobacco products, rather than in dual fashion.

Similar to the United States, SLT manufacturers in South Africa are required by law (since 1995) to include the phrase “causes cancer” on every can of SLT. [13] In recent years, some have however maintained that such federally mandated warnings on SLT products negate harm reduction messages, [14] and that given correct information about the relative harm of SLT products compared to cigarettes, smokers would be motivated towards harm reduction switching. [4] Although Biener et al previously showed that U.S. smokers’ interest in trying low-nitrosamine snus was higher among those who correctly believed it was less harmful than cigarettes, [15] other studies conducted mainly in developed countries suggested that only about one-third or less of smokers expressed interest in using SLT products. [16], [17] Despite these developments, very little information exists on the interest of smokers from low and middle income countries in switching to SLT if informed it is relatively less harmful than cigarettes. To fill this gap in knowledge, this study used nationally representative data from the 2007 South African Social Attitudes Survey (SASAS) to assess smokers’ perceptions on relative harmfulness of SLT and cigarettes, and their interest in harm reduction switching to a hypothetical SLT product if informed it had only a fraction of the risks of cigarettes.

## Methods

### Survey Design/sample

The SASAS is a household survey which uses a multi-stage probability sampling strategy with census enumeration areas as the primary sampling unit. The original protocols for the SASAS were approved by the South African Human Sciences Research Council (HSRC). In 2007, a sample of 3500 households was drawn from the master sample of the HSRC. This SASAS sample was stratified by socio-demographic domain for each province and geographical subtypes, namely tribal areas, formal rural, formal urban and informal urban. This stratification is designed to ensure sufficient geographical distribution across all nine provinces, and adequate distribution between South Africa’s four race groups. From each of the households, one eligible person (≥16 years old) was randomly selected for participation in the survey. [18] In 2007, the pre-tested survey questionnaires were administered by trained interviewers in the respondents’ preferred language, which was selected from among all eleven South African official languages (questionnaires were available in all official languages). The survey was completed by 2907 respondents, yielding an overall response rate of 83.1%.

This study was performed on de-identified secondary data. These data are deposited with the HSRC. While the tobacco use supplement of the SASAS is not publicly available, the data can however be obtained upon special request through the HSRC or its primary collaborator–the University of Pretoria.

### Measures

#### Socio-demographic characteristics

Socio-demographic characteristics assessed included race (Black African; Colored; Indian or Asian; or White); sex (male or female); age (16–24; 25–34; 35–44; 45–54 or ≥55 years), educational attainment (<12; = 12; or >12 years of schooling), marital status (single; married; or widowed/divorced/separated); employment status (unemployed; pensioner/housewife/student; or employed) and region (urban or rural).

#### Tobacco use

Current cigarette smokers were respondents who reported smoking roll-your-own cigarettes or factory-made cigarettes daily or on some days. Current snuff users were respondents who reported using nasal or oral snuff daily or on some days. Data were also collected among smokers on lifetime duration of smoking, number of cigarettes smoked per day (CPD) (1–10; 11–20; 21–30; or >30 cigarettes) and the time from waking up to smoking first cigarette (≤5, 6–30, 31–60, or >60 minutes). We also measured smoking restrictions in the home and work environments.

#### Lifetime quit attempts and quit intentions

**A** lifetime quit attempt among current smokers was defined as any response of “once”, “twice”, “three times or more” to the question: “Have you ever tried to quit smoking?”.

Among current smokers that had made a past quit attempt, methods employed to quit smoking were assessed and included: cold turkey (i.e., just stopped smoking one day without using any smoking cessation aids), cutting-down, use of nicotine replacement therapy (e.g. patch, gum) or prescription medication (e.g. Zyban), or switching to light cigarettes, snuff or snus.

A quit intention was defined as any response of “within the next month”, within the next 6 months”, or “sometime in the future, beyond 6 months” to the question: “Are you planning to quit smoking?” Smokers’ self-efficacy about successfully giving up smoking if they were to try quitting in the next six months was measured on a four-point of “Very likely”; “Fairly likely”; “Not very likely”; or “Not at all likely”.

#### Reasons for smoking and for past quit attempts

Considering that reasons for smoking may influence the level of interest in switching to snuff, smokers were asked reasons why they smoked, which were then categorized into four themes namely; 1) for enjoyment or relaxation; 2) for concentration or coping with daily life; 3) difficulty quitting or lack of willpower to try; and 4) social influences (i.e., helps smoker feel confident around others; perception that smoking is a normal thing to do; or the perception that smoking helps to keep weight down).

Smokers who had made a past quit attempt were also asked what factors motivated them, which were then categorized into six themes namely: 1) cost of smoking; 2) smoking-related health problems or concern for the health of household members; 3) motivation from family and friends; 4) changing social environment towards smoking (i.e., proliferation of smoke-free laws and societal denormalization of smoking); 5) advice from a healthcare professional and; 6) reaction to cigarette health warning labels.

#### Perception about relative harm of snuff compared to cigarettes

Perception about the relative harm of snuff and cigarettes was assessed using the question: “Do you believe that snuff is a safer alternative to smoking cigarettes?” Categorical responses included: “Using snuff is safer than smoking”, “Using snuff is equally as harmful as smoking”, “Using snuff is more harmful than smoking”, and “Do not know”.

#### Interest in switching to snuff

Interest in harm reduction switching to snuff was assessed with the question: “If you currently smoke and were told that snuff is 99% safer than smoking and it would give you the same amount of nicotine you crave from your cigarette, how likely would you be to switch?” Responses of “Very likely” or “Somewhat likely” were categorized together as an affirmative response, while responses of “Somewhat unlikely” or “Very unlikely” were categorized together as a dissenting response.

It is pertinent to note that the survey first asked current smokers about their knowledge of the relative harm of snuff and cigarettes earlier on in the survey and then later asked if they would be interested in switching to snuff if they were told it was 99% safer than cigarettes. Pre-testing of the questionnaire used showed that keeping the phrase ‘99% safer’ also conveyed a local layman’s lingua of ‘confidently’ or ‘surely’, or ‘certainly’ safer.

### Statistical Analysis

Prevalence of current smoking and SLT use were calculated overall and by socio-demographic variables. Comparison of estimates was performed using χ^2^ tests (*p*<0.05).

Level of nicotine dependence among current smokers was assessed using the Heaviness of Smoking Index- a six-point scale calculated from CPD and the time to first cigarette after waking. [19] Scores of 0–1 were categorized as low nicotine dependence; 2–4 as moderate nicotine dependence; and 5–6 as high nicotine dependence. For the purposes of these analyses, moderate (*n = *409) and high (*n = *15) nicotine dependent smokers were analyzed together as one category because of the small sample among the latter.

The primary analysis which assessed smokers’ interest in switching to snuff if informed that snuff was 99% less harmful than cigarettes, was restricted to exclusive cigarette smokers, defined as current smokers who did not report using oral or nasal snuff. However, in a secondary analysis, we also explored the role of snuff in modifying cigarette smoking behavior, by comparing CPD and quit attempts between exclusive cigarette smokers and dual users of cigarettes and snuff.

In order to explore factors independently associated with exclusive smokers’ interest in harm reduction switching, we fitted a multivariate logistic regression model that assessed for presence of smoke-free homes and workplaces, reasons for smoking, heaviness of smoking, lifetime duration of smoking, perceptions about relative harm of snuff and cigarettes, past quit attempts, self-efficacy towards quitting, race, sex, age and education (*p*<0.05). Data were weighted using the “svyset” function in STATA 11 to account for the complex survey design characteristics, and also included the “psu” and “strata” options since the population was sampled by stratifying it first and then randomly selecting several clusters for each stratum.

## Results

### Tobacco Use, Past Quit Attempts and Quit Intentions

Demographic characteristics are shown in **Table** **1**. Overall current smoking prevalence was 20.9% (*n = *689) while the prevalence of current snuff use was 5.0% (*n = *119). Of the current smokers, 97.6% (*n* = 678) exclusively smoked cigarettes, with an average duration of smoking of 13.4 years. About 59.4% (*n = *402) of exclusive cigarette smokers had made a lifetime quit attempt, and of these, 9.4% had tried to quit cigarette smoking by switching to light cigarettes or snuff (Table 2). Among smokers that had made a quit attempt, about 70.0% did so because of concerns about health risks of smoking to themselves or their family members.

**Table 1:** Characteristics of study population, South African Social Attitudes Survey, 2007 (*n* = 2907).

Characteristics	Sample% (n)	Current smoking prevalence* *n* = 689% (95% CI)	Current Snuffing prevalence† *n = *119% (95% CI)	% of current exclusive cigarette smokers ¶ interested in harm reduction switching to snuff *n* = 678
**Race**				
Black African	76.7 (1812)	17.2 (14.8–19.5)	6.5 (4.8–8.2)	32.0 (24.9–39.1)
Colored	9.4 (434)	40.9 (33.2–48.7)	– §	9.3 (4.2–14.4)
Indian or Asian	2.7 (326)	27.6 (18.9–36.3)	– §	17.1 (6.6–27.7)
White	11.2 (335)	28.7 (21.9–35.6)	– §	12.0 (3.4–20.5)
**Sex**				
Male	48.7 (1221)	33 (29.3–36.6)	1.4 (0.6–2.3)	27.7 (21.6–33.7)
Female	51.3 (1686)	9.5 (7.3–11.6)	8.4 (6.1–10.7)	12.8 (7.5–18.0)
**Age, years**				
16–24	27.4 (686)	17.5 (13.6–21.4)	1.2 (0.2–2.2)	16.2 (8.1–24.2)
25–34	26.0 (633)	21.7 (17.4–26.1)	3.3 (1.6–5.0)	27.9 (18.4–37.4)
35–44	17.0 (614)	26.3 (20.9–31.8)	3.6 (0.9–6.3)	24.2 (13.7–34.7)
45–54	13.5 (440)	23.3 (17.3–29.3)	8.7 (5.0–12.4)	36.3 (20.3–52.4)
≥55	16.2 (526)	17.8 (12.9–22.6)	12.7 (7.9–17.5)	17.2 (8.8–25.7)
**Education**				
<12 year of schooling	61.9 (1798)	21.3 (18.5–24.1)	7.2 (5.2–9.2)	28.5 (21.5–35.4)
= 12 year of schooling	28.2 (762)	20.5 (16.3–24.6)	1.1 (0.3–1.9)	13.0 (6.6–19.4)
>12 year of schooling	9.9 (336)	20.7 (14.3–27.0)	1.0 (0.1–2.1)	28.8 (13.6–44.1)
**Marital Status**				
Never married	54.0 (1378)	20.8 (17.7–23.8)	2.8 (1.6–4.0)	24.1 (17.4–30.7)
Widowed, divorced, or separated	12.3 (436)	21.4 (15.0–27.9)	14.7 (8.9–20.4)	18.0 (8.2–27.8)
Married	33.8 (1086)	21.2 (17.9–24.6)	5.2 (3.2–7.2)	26.7 (17.5–35.8)
**Employment**				
Unemployed	32.6 (1037)	29.4 (25.1–33.7)	3.1 (1.8–4.5)	23.3 (15.8–30.8)
Pensioner/student/housewife	44.0 (1177)	19.3 (16.1–22.6)	7.0 (5.0–9.0)	28.6 (20.4–36.8)
Employed	23.4 (680)	12.5 (8.9–16.1)	3.9 (1.3–6.6)	14.7 (4.5–24.9)
**Region**				
Urban	67.5 (2011)	21.7 (18.8–24.5)	4.5 (2.9–6.1)	21.5 (15.5–27.5)
Rural	32.5 (896)	19.4 (15.9–22.9)	6.0 (3.6–8.4)	30.8 (22.2–39.3)
**Overall**	**100.0 (2907)**	**20.9 (18.7–23.1)**	**5.0 (3.7–6.4)**	**24.2 (19.3–29.2)**

**Note**: All samples (*n*) were unweighted while all percentages (%) were weighted to account for the complex survey design. *CI* = Confidence interval.

*Respondents who reported smoking hand-rolled or manufactured cigarettes daily or on some days.

† Respondents who reported using nasal or oral snuff daily or on some days.

§ By race, virtually 100% of current snuffers were Black Africans.

¶ Exclusive cigarette smokers were defined as current smokers that did not report current use of oral or nasal snuff.

**Table 2:** Tobacco use, quit attempts and perceptions of current exclusive cigarette smokers, South African Social Attitudes Survey, 2007.

Characteristic	Overall * *n* = 678Estimate (95% CI)	Males *n* = 451Estimate (95% CI)	Females *n = *227Estimate (95% CI)
*Smoking characteristics among all current exclusive cigarette* *smokers (n = 678)*			
**Mean number of cigarettes smoked per day, cigarette sticks**	9.5 (8.6–10.4)	9.1 (8.1–10.2)	10.6 (8.8–12.3)
**Mean duration of smoking, years**	13.4 (12.1–14.8)	13.0 (11.5–14.5)	14.9 (11.9–17.9)
**Reasons for smoking, %**			
For enjoyment or relaxation	74.0 (69.4–78.6)	73.1 (67.6–78.5)	77.2 (68.7–85.7)
For concentration or to cope with daily life	29.2 (24.2–34.2)	29.4 (23.7–35.0)	28.5 (18.7–38.2)
Difficulty quitting or lack of willpower to try	32.4 (26.8–38.0)	31.5 (24.8–38.2)	35.3 (25.9–44.6)
Social influences	20.0 (15.6–24.4)	18.9 (14.0–23.7)	24.0 (16.0–32.1)
*Past Quit attempts among current exclusive cigarette smokers that had* *ever made a quit attempt (n = 402)*			
**% of current smokers that had ever made a quit attempt**	59.4 (53.6–65.3)	59.6 (52.6–66.6)	59.0 (48.9–69.0)
**Number of past quit attempts, %**			
1	41.8 (34.0–49.6)	45.1 (35.9–54.4)	31.0 (20.4–41.4)
2	20.6 (15.5–25.7)	20.2 (14.1–26.2)	22.0 (12.9–31.0)
3+	37.6 (30.6–44.6)	34.7 (26.5–42.9)	47.1 (36.3–57.8)
**Method used in past quit attempts, %**			
Cold turkey (i.e., just stopped smoking one day)	22.5 (16.9–28.1)	20.9 (14.3–27.5)	27.4 (16.8–38.0)
Cutting down	59.7 (52.7–66.7)	62.0 (53.5–70.5)	52.7 (42.1–63.3)
Switching to light cigarettes or smokeless tobacco	9.4 (4.7–14.0)	8.7 (3.0–14.5)	11.3 (4.0–18.6)
Counseling or pharmacotherapy †	8.4 (4.4–12.4)	8.3 (3.6–13.1)	8.6 (1.1–16.0)
**Reasons for past quit attempts, %**			
Cost of smoking	44.3 (36.4–52.2)	41.3 (32.0–50.5)	54.5 (41.0–68.3)
Health concerns	70.0 (63.2–76.8)	65.7 (57.6–73.9)	84.6 (77.2–92.0)
Motivation from family and friends	12.8 (7.8–17.7)	13.8 (7.7–19.9)	9.2 (3.9–14.4)
Changing social environment towards smoking	13.9 (8.1–19.7)	13.7 (6.9–20.5)	14.6 (3.6–25.6)
Advice from a healthcare professional	5.5 (2.5–8.4)	4.8 (1.4–8.2)	7.9 (2.1–13.7)
Reaction to cigarette health warning label	5.0 (2.0–8.0)	5.5 (1.8–9.3)	3.1 (0.1–6.5)
*Quit intentions among all current exclusive cigarette smokers (n = 678)*			
**% with quit intentions**	52.8 (46.5–59.1)	50.9 (43.6–58.3)	59.3 (47.8–70.9)
**% of those intending to quit who felt confident of at least a** **fair chance at success if they tried quitting in the next 6 months** §	62.0 (54.9–69.1)	62.5 (51.7–73.3)	62.0 (52.3–71.7)
*Perceptions about relative harm of tobacco products among* *all current exclusive cigarette smokers (n = 678)*			
**Perception of the relative harm of snuff compared to cigarettes, %**			
Using snuff is safer than smoking	5.7 (3.4–8.0)	5.6 (2.9–8.3)	6.2 (2.0–10.4)
Using snuff is as equally harmful as smoking	49.7 (44.1–55.2)	51.7 (45.0–58.4)	42.8 (33.9–51.7)
Using snuff is more harmful than smoking	12.9 (9.5–16.2)	12.1 (8.3–15.9)	15.5 (8.2–22.8)
Did not know	31.8 (26.4–37.1)	30.7 (24.3–37.0)	35.5 (26.8–44.3)

**Note**: All samples (*n*) were unweighted while all percentages (%) were weighted to account for the complex survey design. *CI* = Confidence interval.

*Some totals may add up to over 100% because multiple responses were allowed.

† Counseling included individual and group counseling sessions, including those by a faith/religious or traditional healer. Pharmacotherapy included use of nicotine replacement therapy (e.g. patch, gum) as well as prescription medication (e.g. Zyban).

§ Respondents who thought they were “fairly likely” or “very likely” at being successful at quitting if they attempted within the next 6 months.

There were no significant difference observed in the proportion of past-quit-attempters between exclusive cigarette smokers (59.4%) and dual users of cigarettes and snuff (68.5%). Similarly, the average CPD among exclusive cigarette smokers (mean = 9.5; standard deviation = 9.1) did not vary significantly from dual users of cigarettes and snuff (mean = 8.4; standard deviation = 5.6).

### Perception about Relative Harm of Snuff Compared to Cigarettes

The proportion of respondents who believed that using snuff is equally as harmful as smoking cigarettes was significantly higher among exclusive cigarette smokers (49.7%) compared to snuffers (21.8%) (*p*<0.05). In contrast, the proportion that believed that using snuff is safer than smoking was significantly lower among exclusive cigarette smokers (5.7%) compared to snuffers (67.5%) (*p*<0.05). Also, 31.8% of exclusive cigarette smokers versus 2.7% of snuffers reported not knowing if there was a difference in harm between snuff and cigarettes (*p*<0.05). No significant smoker-snuffer difference was observed in the proportion who believed that snuff is more dangerous than cigarettes (12.9% vs. 8.0% respectively; Table 3).

**Table 3:** Comparative risk perception between non users of tobacco, current cigarette smokers and current snuffers, South African Social Attitudes Survey, 2007.

Perception	Non-tobacco users* *n = *1991% (95% CI)	Current exclusivecigarette smokers† *n = *678% (95% CI)	Current snuffers§ *n = *119% (95% CI)	% of exclusive cigarette smokers interested in switching to snuff if informed it was 99% safer than cigarettes and yielded the same amount of nicotine as cigarettes (95% CI)
Using snuff is safer than smoking	11.9 (9.7–14.2)	5.7 (3.4–8.0)	67.5 (56.4–78.5)	26.2 (9.1–43.3)
Using snuff is as equally harmfulas smoking	48.5 (44.9–52.1)	49.7 (44.1–55.2)	21.8 (11.8–31.7)	27.7 (19.6–35.8)
Using snuff is more harmful than smoking	10.0 (8.0–11.9)	12.9 (9.5–16.2)	8 (0.3–15.8)	34.7 (21.9–47.5)
Did not know	29.6 (26.3–33.0)	31.8 (26.4–37.1)	2.7 (0.0–5.7)	14.5 (7.8–21.2)

**Note**: All samples (*n*) were unweighted while all percentages (%) were weighted to account for the complex survey design. *CI* = Confidence interval.

*Respondents that had never used any of the following tobacco products during their lifetime: manufactured cigarettes, hand-rolled cigarettes, pipes or cigars or smokeless tobacco products (including nasal or oral snuff).

† Respondents who reported smoking roll-your-own cigarettes or factory-made cigarette daily or some days, but who were not current users of oral or nasal snuff.

§ Respondents who reported using oral or nasal snuff daily or some days.

### Interest in Harm Reduction Switching

About 24.2% of all exclusive cigarette smokers indicated interest in harm reduction switching to snuff. This proportion was highest (34.7%) among exclusive cigarette smokers who believed a priori that snuff is more harmful than cigarettes, and lowest (14.5%) among those who did not know if there was a difference in harm between snuff and cigarettes. After adjusting for all other factors, this latter group remained less likely to be interested in harm reduction switching (adjusted odds ratio [*a*OR] = 0.42; 95% confidence interval [CI]: 0.19–0.91). Other factors associated with interest in harm reduction switching included: presence of 100% smoke-free policies at work (*a*OR = 2.05; 95%CI: 1.09–3.97); increasing self-efficacy towards successfully quitting (*a*OR = 1.49; 95%CI: 1.21–1.84) and smoking cigarettes primarily to enhance concentration or cope with everyday life (*a*OR = 1.58; 95%CI: 1.02–2.46). History of at least one past quit attempt was associated with lowered interest in switching to snuff (*a*OR = 0.58; 95%CI: 0.35–0.95) (Table 4).

**Table 4:** Logistic regression analyses of factors associated with interest in harm reduction switching to snuff among current exclusive cigarette smokers (*n* = 678), South African Social Attitudes Survey, 2007.

Characteristic	Categories	Crude odds ratios,95% CI	Adjusted odds ratios,95% CI
**Smoke-free environments**			
Smoke-free home regulations	Absent (Referent)		
	Present	1.60 (1.12–2.29)	1.35 (0.83–2.20)
Smoke-free policies at work	No smoke-free policy at work (Referent)		
	Partial bans on workplace smoking	1.24 (0.72–2.14)	1.30 (0.65–2.59)
	100% smoke-free policies at work	2.14 (1.35–3.39)	2.05 (1.09–3.87) *
	Did not know/did not answer	0.71 (0.38–1.34)	1.14 (0.52–2.49)
**Self-reported reasons for Smoking**			
For pleasure or enjoyment (i.e., hedonistic reward)	No (Referent)		
	Yes	0.43 (0.27–0.69)	0.67 (0.38–1.17)
To enhance concentration or cope with everyday life	No (Referent)		
	Yes	1.74 (1.19–2.54)	1.58 (1.02–2.46) *
Difficulty quitting or lack of willpower to try	No (Referent)		
	Yes	0.66 (0.45–0.98)	1.20 (0.74–1.93)
Social influences †	No (Referent)		
	Yes	0.84 (0.53–1.33)	0.79 (0.46–1.34)
**Tobacco use characteristics and perception**			
Heaviness of smoking index	Low dependence (Referent)		
	Moderate to heavy dependence	0.62 (0.43–0.91)	0.76 (0.48–1.21)
Duration of smoking, years	(Per unit increase)	1.00 (0.98–1.02)	1.02 (0.99–1.05)
Perception of relative harm from snuff use	Snuff is safer than smoking (Referent)		
	Snuff equally harmful as smoking	0.76 (0.40–1.44)	0.64 (0.31–1.32)
	Snuff more harmful than smoking	1.00 (0.49–2.06)	1.04 (0.43–2.52)
	Did not know	0.39 (0.20–0.76)	0.42 (0.19–0.91) *
**Lifetime Quit attempts**			
Past quit attempt	None (Referent)		
	≥1	0.79 (0.55–1.13)	0.58 (0.35–0.95) *
Number of past quit attempts(among past quit attempters)	1 (Referent)		
	2	0.51 (0.20–1.28)	0.47 (0.18–1.20)
	≥3	0.33 (0.14–0.75)	0.30 (0.12–0.71)
Self efficacy to quit ¶	(Per unit increase)	1.43 (1.20–1.70)	1.49 (1.21–1.84) *
**Socio-demographic characteristics**			
Race	Black African (Referent)		
	Colored	0.38 (0.22–0.64)	0.48 (0.23–1.02)
	Indian or Asian	0.39 (0.21–0.72)	0.59 (0.28–1.27)
	White	0.30 (0.15–0.59)	0.57 (0.22–1.43)
Gender	Male (Referent)		
	Female	0.54 (0.36–0.80)	0.75 (0.43–1.30)
Age, years	16–24 (Referent)		
	25–34	1.29 (0.75–2.20)	1.59 (0.80–3.19)
	35–44	0.94 (0.53–1.66)	1.10 (0.51–2.39)
	45–54	1.39 (0.78–2.48)	1.67 (0.73–3.83)
	≥55	1.01 (0.56–1.82)	1.00 (0.34–2.95)
Education	<12 year of schooling (Referent)		
	= 12 year of schooling	0.58 (0.36–0.93)	0.57 (0.31–1.03)
	>12 year of schooling	0.69 (0.36–1.33)	0.69 (0.33–1.47)

**Note**: All analyses were weighted to account for the complex survey design. CI = Confidence interval.

*Statistical significant after adjusting for all other factors listed in table (*P*<0.05).

† Social influences included responses by participants that they smoked to keep their weight down, for confidence or because of peer influence.

¶ Measured on a Likert-type scale consisting of the responses: “Very likely”, “Fairly likely”, “Not very likely”, and “Not at all likely”.

## Discussion

Similar to findings in the literature, [20] this study demonstrated that about a quarter of exclusive cigarette smokers indicated interest in harm reduction switching to SLT products. The fact that only a relatively small fraction of smokers were interested in harm reduction switching may attest to the fact that while nicotine is the primary addictive agent in tobacco, the role of smoking sensory experience (e.g., taste, flavor, and mouth-feel of tobacco smoke), as well as behavioral addictions (e.g., the hand-to-mouth motions of smoking) as secondary reinforcers of smoking behavior cannot be under-estimated. Thus, a lack of interest among smokers in switching to SLT products may not necessarily indicate a lack of awareness about the risks of smoking. For example, among smokers that had made a quit attempt, 70% had done so out of concern for smoking related health problems, indicating that ignorance about the health risks of smoking is not likely an issue among this population. Taken together, this might suggest that promotion of snuff use among this predominant group of smokers not interested in harm reduction switching may only encourage dual use of cigarettes and SLT (i.e. using SLT only where smoking is not allowed), which might cause net population harm, especially if it reduces the impact of smoke-free laws in promoting tobacco cessation. [8] This is particularly pertinent in the studied population considering that a significantly higher proportion of exclusive cigarette smokers with 100% smoke-free policies at work indicated interest in harm reduction switching.

The likelihood of harm reduction switching was significantly lower among current exclusive smokers that had made a quit attempt, and further reduced with the number of past quit attempts. In other words, the use of SLT as a substitute to smoking seemed to be attractive to those who might be considered inveterate smokers. Indeed, previous studies have suggested that interest in SLT was greater among smokers who were unwilling to make a quit attempt, [21] or were not planning to quit smoking. [16] The observation of those that had made multiple quit attempts not being interested in switching may suggest that these past quit-attempters may be more interested in attaining and maintaining a tobacco-free status rather than harm reduction switching to another tobacco product. This may also explain why the use of snuff or “light cigarettes” as a smoking cessation aid was also relatively unpopular among smokers that had made a past quit attempt (9.4%). Even among smokers that had successfully quit smoking (i.e., former smokers, *n = *75), only 5.4% had ever used snuff, while the majority had done so using ‘cold turkey’ (data not shown). These findings are corroborated from a recent study among Nigerian male smokers which showed no significant difference in smoking intensity among exclusive cigarette smokers when compared to current smokers that also used snuff, indicating that snuff use was not associated with reduced harm among continuing smokers who had tried snuff. [22] The fact that exclusive smokers in our study who expressed greater self-confidence in quitting were also more likely to express a greater interest in switching indicates that smokers’ self-efficacy at baseline is a potential confounder that should be controlled for in future studies on the effect of snuff on quitting smoking.

It is pertinent to note that the majority of snuff users (67.5%), although exposed to the same health warnings as cigarette smokers, were more likely to believe that snuff was less harmful than cigarettes. This suggests that the observed differences in belief by tobacco use status might partly reflect a social judgment rather than a true perceived relative risk. It is conceivable that users of a particular product that is considered socially undesirable may want to justify continued use by believing their product to be no more harmful than another. The alternative explanation though is that snuff users use it because they correctly believe it to be safer than cigarette smoking. The cost savings on switching to SLT products may be a further incentive, especially for price-sensitive smokers, as traditional SLT products in South Africa are about six times cheaper than premium brand cigarettes ($0.50 for 20 g SLT, vs. $3 for a pack of 20 cigarettes). However, this relatively lower cost of SLT products could also be an incentive for young never tobacco users to initiate tobacco use.

While the question posed to respondents asked them to assume that the hypothetical SLT product was 99% safer than cigarettes, this may be an oversimplification of the reality of the relative harm of SLT products. Although the body of scientific evidence shows that SLT products contain relatively lower levels of nitrosamines and other toxins compared to combustible tobacco products, this in no way implies that SLT products are harmless or a safe alternative to cigarettes, especially so for the traditional products that are more affordable in low and middle income countries. [2] SLT use has been associated with several malignancies including oral and esophageal cancers. [23] More so, given that several multi-national cigarette companies have invested significantly in SLT manufacture and several cigarette-branded SLT products are available, [24] the potential of the tobacco industry promoting dual use rather smoking cessation needs to be given consideration. This is important considering that dual use among current cigarette smokers is a significant public health problem, with over a fifth of cigarette smokers in 28 of 44 countries assessed in a recent study reporting concurrent use of at least one other non-cigarette tobacco product. [25] Interestingly, our study showed that while Colored respondents (i.e. those of mixed ancestry) as well as Whites had the highest smoking prevalence, they paradoxically had the lowest proportion indicating interest in harm reduction switching to snuff. This may suggest that the Swedish experience may not be transferrable to societies without a strong SLT culture or those with unique tobacco use habits or patterns. [3].

This study has some limitations. First, we did not make a distinction as to the application route of this hypothetical reduced harm SLT product. However, given that the aim was to explore the interest of South African smokers in switching to a considerably less harmful product and considering that the level of interest in harm reduction switching in this study was comparable to that reported in similar studies elsewhere; [20] it is likely that the conclusions reached in this study will not differ even if the route of application had been specified. Second, self-reported tobacco use status and the cross-sectional study design may have resulted in a mis-reporting of tobacco use and may also preclude drawing causal inferences. Third, the fact that smokers expressed interest in switching to snuff does not necessarily mean they may actually switch–especially inveterate smokers or heavy smokers–considering the interplay between neurobiological and behavioral factors in smoking addiction. Fourth, it is possible that the phrasing of our survey question which suggested complete switching to cigarettes may not capture smokers who may not wish to switch completely, but rather be interested in SLT only to reduce their smoking intensity. Finally, since data were collected in 2007, it is possible that smokers’ views or perceptions may have changed.

## Conclusion

This study demonstrated that about a quarter of South African exclusive cigarette smokers sampled in a 2007 population-based survey indicated interest in harm reduction switching to snuff. SLT products have a potential role in reducing the harm from cigarettes smoking but only if they are not used to circumvent smoke-free laws that have been associated with significant reductions in smoking prevalence.
